# Effectiveness of a Blended Multidisciplinary Intervention for Patients with Moderate Medically Unexplained Physical Symptoms (PARASOL): Protocol for a Cluster Randomized Clinical Trial

**DOI:** 10.2196/resprot.9404

**Published:** 2018-05-08

**Authors:** Paula Elisabeth van Westrienen, Martijn F Pisters, Suze AJ Toonders, Marloes Gerrits, Cindy Veenhof, Niek J de Wit

**Affiliations:** ^1^ Department of Health Innovation and Technology Fontys University of Applied Sciences Eindhoven Netherlands; ^2^ Center for Physical Therapy Research and Innovation in Primary Care Utrecht Netherlands; ^3^ Physical Therapy Research Department of Rehabilitation, Physical Therapy Science and Sport, Brain Center Rudolf Magnus University Medical Center Utrecht Utrecht Netherlands; ^4^ Department of General Practice Julius Center for Health Sciences and Primary Care University Medical Center Utrecht Utrecht Netherlands; ^5^ Research Group Innovation of Human Movement Care University of Applied Sciences Utrecht Utrecht Netherlands

**Keywords:** medically unexplained physical symptoms, blended care, multidisciplinary, primary care, intervention

## Abstract

**Background:**

Medically unexplained physical symptoms are an important health problem in primary care, with a spectrum from mild to chronic. The burden of chronic medically unexplained physical symptoms is substantial for patients, health care professionals, and society. Therefore, early identification of patients with moderate medically unexplained physical symptoms is needed in order to prevent chronicity. The preventive screening of medically unexplained physical symptoms (PRESUME) screening method was developed using data from the electronic medical record of the patients' general practitioner and demonstrated its prognostic accuracy to identify patients with moderate medically unexplained physical symptoms. In the next step, we developed a proactive blended and integrated mental health and physical therapy intervention program (PARASOL) to reduce complaints of moderate medically unexplained physical symptoms, stimulate self-management, and prevent chronicity.

**Objective:**

The primary objective of this study is to investigate the effectiveness of the blended PARASOL intervention on the impact of symptoms and quality of life in patients with moderate medically unexplained physical symptoms compared with usual care. Secondary objectives are to study the effect on severity of physical and psychosocial symptoms, general health, physical behavior, illness perception, and self-efficacy in patients with moderate medically unexplained physical symptoms as well as to determine the cost-effectiveness of the program.

**Methods:**

This paper presents the study protocol of a multicenter cluster randomized clinical trial. Adult patients with moderate medically unexplained physical symptoms will be identified from electronic medical record data using the PRESUME screening method and proactively recruited for participation in the study. Cluster randomization will be performed at the level of the participating health care centers. In total 248 patients with moderate medically unexplained physical symptoms (124 patients per arm) are needed. The PARASOL intervention is a 12-week blended primary care program consisting of 4 face-to-face consultations with the mental health nurse and 5 physical therapy sessions, supplemented with a Web-based program. The Web-based program contains (1) information modules and videos on self-management and educative themes, (2) videos and instructions on prescribed home exercises, and (3) assignments to gradually increase the physical activity. The program is directed at patients’ perception of symptoms as well as modifiable prognostic risk factors for chronicity using therapeutic neuroscience education. It encourages self-management, as well as an active lifestyle using a cognitive behavioral approach and graded activity. Primary outcomes are impact of symptoms and quality of life. Secondary outcomes are severity of physical and psychosocial symptoms, general health, physical behavior, illness perceptions, self-efficacy, and cost-effectiveness. All measurements will be performed at baseline, 3 and 12 months after baseline. Retrospective cost questionnaires will also be sent at 6 and 9 months after baseline and these will be used for the cost-effectiveness analysis.

**Results:**

The intervention has been developed, and the physical therapists and mental health nurses in the participating experimental health care centers have received two days of training on the content of the blended PARASOL intervention. The recruitment of health care centers started in June 2016 and inclusion of patients began in March 2017. Follow-up assessments of patients are expected to be completed in March 2019.

**Conclusions:**

This study is the first randomized clinical trial to determine the effectiveness (including cost-effectiveness) of a proactive, blended, and integrated mental health and physical therapy care program for patients with moderate medically unexplained physical symptoms. The findings will help to improve the treatment for patients with moderate medically unexplained physical symptoms and prevent chronicity.

**Trial Registration:**

Netherlands Trial Register NTR6755; http://www.trialregister.nl/trialreg/admin/rctview.asp?TC=6755 (Archived by WebCite at http://www.webcitation.org/6ywporY7u).

## Introduction

Medically unexplained physical symptoms (MUPS), especially pain, dizziness, and fatigue are frequent in primary care, in fact 25%-50% of all symptoms presented during consultations cannot be adequately medically explained [1]. If there are physical complaints for which no medical condition can be found after adequate medical examination, they will be defined as MUPS [2,3].

MUPS can be regarded as a spectrum ranging from mild unexplained physical symptoms (low incidence, one or two domains, low impact), to moderate symptoms (more frequent, two or three domains, higher impact) and finally to persisting or chronic MUPS (high impact, more clusters involved, chronic; eg, fibromyalgia, chronic fatigue syndrome, or irritable bowel syndrome) [3,4]. In this spectrum, mild MUPS have an estimated prevalence of 70% to 80% [4,5]. These patients consult their general practitioner (GP) for a symptom that cannot be explained immediately, but the symptoms improve within 2 weeks [6]. Moderate MUPS have an estimated prevalence of approximately 15%, where patients still experience unexplained symptoms after three months without a diagnosis of a functional somatic syndrome [6]. Patients with chronic MUPS will have a symptom duration of at least six months, with the presence of a functional somatic syndrome, such as fibromyalgia, chronic fatigue syndrome or irritable bowel syndrome, or a somatic symptom disorder according to the Diagnostic and Statistical Manual of Mental Disorders, 5th edition [4,6,7]. Patients with chronic MUPS occur in approximately 2.5% in primary care, and 3% of the GP consultations are MUPS consultations [1,8].

Despite the low prevalence of chronic MUPS, the burden is substantial [1], with a high impact on patients’ quality of life and daily functioning. Compared with the general population, as well with other patient groups such as major depressive disorder and cancer patients, patients with chronic MUPS report a lower quality of life [9,10]. Moreover, patients with MUPS consult a GP more frequently, but GPs find adequate management of MUPS challenging [11].

GPs frequently focus on exclusion of a somatic disease by recommending somatic interventions such as drug prescriptions, an investigation or a referral to a specialist; while patients often do not request for somatic interventions [12]. Furthermore, GPs face difficulty in the timely recognition of patients with MUPS [13]. On average, it takes two years to obtain a diagnosis. During this time period patients have on average 15 GP consultations, 8 visits to a hospital specialist and 14 sessions with the physical therapist [10]. Almost 40% of patients with MUPS report absenteeism from work [10]. As a result, MUPS are associated with increased direct and indirect costs related to health care expenditure as well as work and insurance related costs [10,14].

Much research has been conducted on effective interventions for chronic MUPS. Neurosciences-based therapeutic education, cognitive behavioral therapy, and exercise therapy have been shown to be effective treatment modalities in patients with MUPS [15-18]. Overall, the vast majority of these studies included patients with chronic MUPS. So far little research has been conducted in patients with moderate MUPS, partly due to the fact that adequate methods for early identification are lacking. Early identification of patients with moderate MUPS would enable interventions directed at prevention of chronicity, which ultimately might decrease the burden of these symptoms for patients, health care professionals and society.

**Figure 1:**
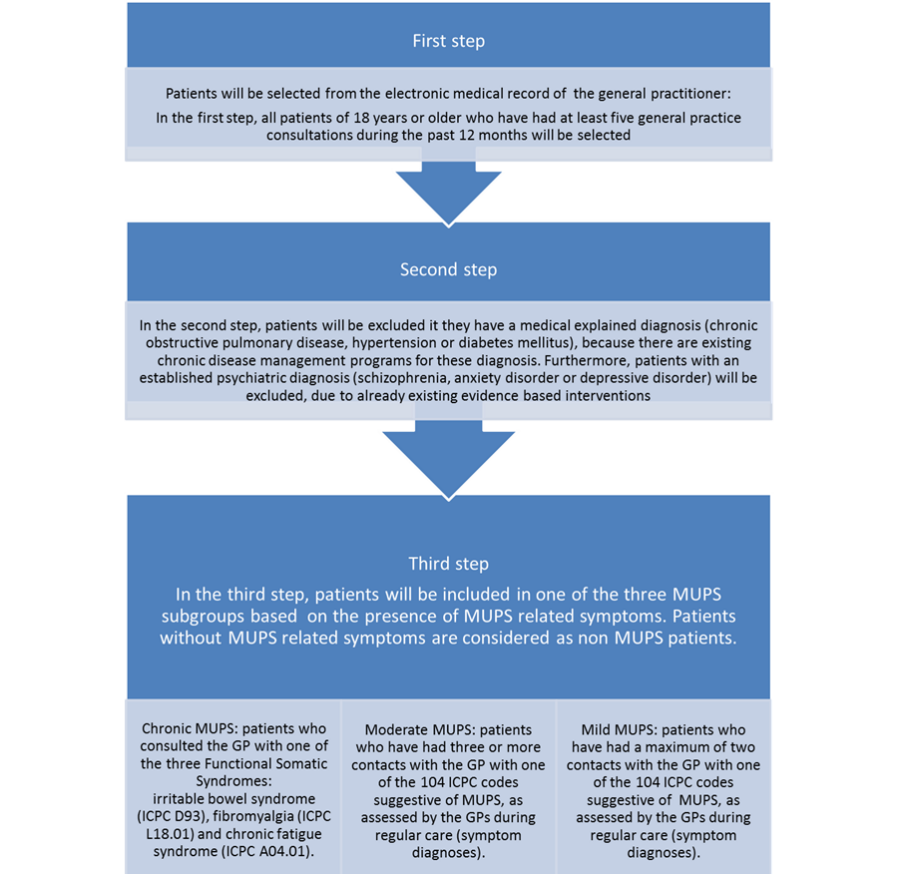
PRESUME screening method.

Recently, a screening method (PRESUME; preventive screening of medically unexplained physical symptoms) has been developed to identify patients with moderate MUPS using data from the electronic medical record of the patient’s GP as shown in Figure 1 [19]. The PRESUME screening method showed acceptable prognostic accuracy over a five-year follow-up [19]. For patients with moderate MUPS, we developed a proactive, blended, and integrated mental health and physical therapy care program to prevent chronicity. This is a 12-week program consisting of 4 face-to-face consultations with the mental health nurse and 5 physical therapy sessions, which are supplemented with a Web-based program (e-Exercise). Blended care has already proven to be effective in other studies [20,21] and it helps to promote self-management.

The primary objective of the present study is to investigate the effectiveness of the proactive, blended and integrated mental health and physical therapy care program (PARASOL) on impact of symptoms, as well as the physical and mental dimensions of quality of life in patients with moderate MUPS in comparison with usual care. Secondary objectives are to study the effect on severity of (psychosocial) symptoms, general health, physical behavior, illness perception, and self-efficacy in patients with moderate MUPS as well as to determine the cost-effectiveness of this program.

## Methods

### Study Design

**Figure 2:**
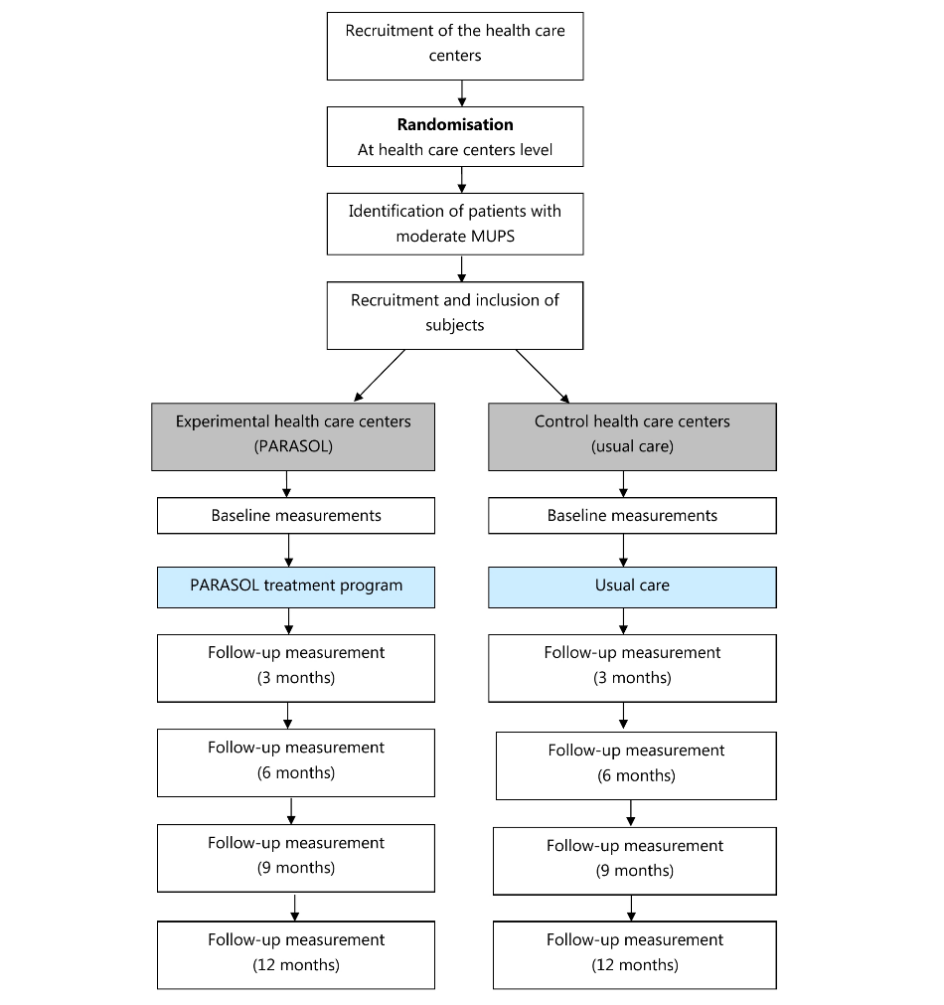
Overview of the study.

A prospective, multicenter cluster randomized clinical trial will be conducted. The study has been approved by the Medical Ethical Committee of University Medical Center Utrecht, the Netherlands. The blended PARASOL intervention will be compared with usual care. An overview of the study procedure is shown in Figure 2.

### Participants

#### Patient selection

Patients with moderate MUPS will be identified in the participating practices using 3 strategies. The first strategy is to use the PRESUME screening method. All patients in the routine care database of a GP are anonymously screened in a stepwise selection, based on a consultation frequency above five, with exclusion of chronic diseases (eg, chronic obstructive pulmonary disease, hypertension or diabetes mellitus) and psychiatric diagnoses (eg, schizophrenia, anxiety disorder or depressive disorder) and the presence of any of the 104 MUPS related International Classification of Primary Care codes. The prognostic accuracy of this PRESUME screening method for identification of moderate MUPS patients is moderate [19].

Since the PRESUME screening method is over inclusive and not meant to set an accurate diagnosis of MUPS in individual patients, all identified patients with moderate MUPS will be screened by their GP for eligibility [19]. As a consequence, the expected prevalence of patients with moderate MUPS is less than the 2.4% according to the PRESUME screening method [19]. The GP will exclude patients based on the following criteria: (1) having another chronic somatic or psychiatric disease, (2) receiving a medically explained diagnosis between identification using the PRESUME screening method and the time of inclusion, (3) having complaints with a duration of less than 1 month, in which case further diagnostic evaluation of the symptoms is needed, and (4) unable to participate as determined by the GP, due to a life-threatening condition, a shortened life expectancy, a major life event in the past month or a MUPS targeted multidisciplinary intervention in the past 12 months.

All remaining eligible patients will proactively be approached by their GP, by sending them an invitation letter with study information.

Secondly, GPs will recruit patients during consultations if they meet the following criteria: ≥18 years of age, ≥5 general practice consultations during the past twelve months, medically unexplained physical symptoms, and the diagnostic phase is completed. When a patient is eligible, the GP can give the contact details of the researchers of the PARASOL study to the patient.

The last strategy will be open recruitment in participating health care centers. Flyers with information about the PARASOL study will be provided in the waiting rooms and included in the newsletter of the health care centers. Patients who are willing to participate can contact the researcher by phone or by mail. Subsequently, the researcher will determine whether the patient is eligible by asking if the patient is older than 18 years, has had ≥5 general practice consultations during the past twelve months, and if the patient has medically unexplained physical symptoms.

All patients who are willing to participate in the PARASOL study, will have to have access to the internet and have mastered the Dutch language. When a patient is willing to participate, they can contact the researcher by phone or email. The researcher will answer any possible questions, give further information, and will make an appointment for the patient to sign informed consent and a baseline measurement evaluation. Additionally, patients in the intervention group will be invited to participate in the blended PARASOL intervention.

#### Study centers

The Leidsche Rijn Julius Health Care Centers (LRJG; 5 health care centers with 40,000 patients) and the Eindhoven Corporation of Primary Health Care Centers (SGE; 10 health care centers, 70,000 patients) will participate in the study. All relevant disciplines—general practitioners, physical therapists, and mental health nurses—are available and willing to participate.

### Randomization Procedure

Cluster randomization will be performed at the level of the participating health care centers. Health care centers will randomly be assigned to either the intervention group or the control group (usual care) using a Web-based random generation of a sequence of numbers. Through cluster randomization, we will avoid professionals within one health care center offering both the blended PARASOL intervention and usual care, as this could cause potential contamination effects [22]. A higher drop-out rate in the intervention group is expected since psychological therapies have a 7% higher proportion of drop outs compared with usual care [18]. The blended PARASOL intervention combines both mental health and physical therapy sessions. Therefore, an unequal randomization on cluster level will be conducted. Of the 15 included health care centers, 8 will be randomized to the blended PARASOL intervention and 7 will be randomized to the control group. After randomization of the health care centers, the selection and inclusion procedure of patients with moderate MUPS will be performed.

### Intervention Program

The health care program is a proactive, blended, and integrated care program offered by a physical therapist and mental health nurse. The program will start with a physical approach since patients’ perception of the symptoms usually has a somatic focus and MUPS patients are often reluctant to accept psychological oriented treatments [23,24]. The aim of the health care program is to reduce complaints of moderate MUPS, stimulate self-management, and prevent chronic MUPS. The health care program is focused on patients’ insight, perception of symptoms, and modifiable prognostic risk factors for the development of chronic MUPS, using a cognitive behavioral approach and therapeutic neuroscience education as well as encouraging self-management and an active lifestyle using graded activity (details are provided in Multimedia Appendix 1). It consists of 3 steps and the face-to-face sessions will be integrated with eHealth modules, called blended health care. The content of the eHealth modules will be discussed during the face-to-face sessions. Details of the 3 steps are listed below:

1. Intake: The program will start with an intake session with both the physical therapist and the mental health nurse. During the intake session the complaints, treatment goals, treatment demand, and perpetuating factors of the patient will be identified according to the somatic, cognitive, emotional, behavioral, and social factors (SCEGS) model [3]. After the intake the physical therapist and mental health nurse discuss the complaints, treatment goals, and treatment demand.
  - The physical therapist will focus on the somatic complaints (ie, physical symptoms, duration and course of symptoms, severity of symptoms, and physical functioning) and will conduct a physical examination to get insight to factors that are related to the content of the health care program (eg, posture and movement, breathing patterns, and muscle tension) and to determine if symptom specific exercises are needed.
  - The mental health nurse will focus on cognitive, emotional, behavioral and social complaints.
2. Face-to-face sessions:
  - Patients will have 4 face-to-face sessions with the physical therapist (week 1, week 3, week 6 and week 12) where the focus will be on the perception and acceptation of physical complaints of the patients. The physical therapist will start with education regarding the unexplained symptoms. Therapeutic neuroscience education according to the sensitization model is of particular interest due to patient’s somatic fixation and anxiety for a severe disease [17]. Concurrently, graded activity will be used to gradually expand activities performed by the patient using principles of operant conditioning [25,26]. The graded activity schedule can be performed in daily life. In week 6, the physical therapist will discuss the patients’ lifestyle (eg, exercise, sleep, and relaxation) with the focus on behavioral changes to promote a healthy lifestyle. In week 12, the physical therapist will discuss long-term goals as well as how patients can maintain a physically active lifestyle.
  - Patients will have 3 face-to-face sessions with the mental health nurse (week 1, week 3, and week 6). In all 3 face-to-face sessions the mental health nurse will train coping strategies according to perpetuating factors and operant conditioning [25], with the focus on changing perception and acceptation. The mental health nurse will start with education regarding general perpetuating factors with the link to possible perpetuating factors of the patient. In the next 2 face-to-face sessions, the link between the perpetuating factors and patients coping strategies will be made, with the focus on behavioral change.
3. eHealth modules: The Web-based part of the health care program consists of exercises (instruction videos) and information modules on self-management and educative themes (description and videos). The modules consist of 3 components which are listed below.
  - Graded activity, an activity-focused method with operant conditioning behavioral principles with 3 consecutive phases. In the starting phase, the patient will choose an activity they want to expand gradually. The patient will perform the chosen activity to their tolerance level (ie, until pain or fatigue drives them to stop; this will be pain-contingent) while their performance is recorded in distance units, time, or number of repetitions. After at least 3 pain-contingent measurements, occurring over several days, a baseline will be determined, and the patient sets his or her individual treatment goal. In the treatment phase, the chosen activity will be increased gradually (ie, time-contingent) and an individual scheme will be drawn up. In the integration phase, patients will be stimulated to adhere to the activity in their daily living [25,26]
  - Videos of prescribed home exercises by their physical therapist
  - Videos and information on self-management and educational themes such as central sensitization, perpetuating factors, graded activity, behavioral change, stress, coping, relaxation, lifestyle advice, creating and performing an exercise plan, and avoiding a relapse.

### Usual Care

Patients in the control health care centers will get care as per usual without any restrictions. This care could include care of the GP, physical therapist, mental health nurse, and psychologist.

### Outcomes

#### Primary Outcome

The primary outcome measures are impact of symptoms and quality of life.

#### Secondary Outcomes

Several secondary parameters will be measured to determine the influence of the blended e-Exercise health care program on severity of physical and psychosocial symptoms, general health, physical behavior, illness perceptions, self-efficacy, and cost-effectiveness.

#### Measurements

Three time points (baseline, 3-month, and 12-month follow-up) will be used for data collection. In addition, cost questionnaires will also be sent to the patients at 6 and 9 months. Furthermore, the impact of symptoms will be measured weekly between 0 and 3 months, followed by monthly measurements between 6 and 12 months. We offer no financial incentives to complete questionnaires or to carry the Acitv8 activity monitor. The measures that will be collected are listed below and Table 1 gives a summary of all measures that will be collected.

- Impact of symptoms, which addresses adequate relief using a validated single question, which is scored on a dichotomous scale (“Over the past week have you had adequate relief of your symptoms?”) [27,28]. A responder for adequate short-term relief is defined as a patient who will report adequate relief of their symptoms for at least six of the twelve weeks between the baseline and three-month follow-up. In addition, a responder for adequate long-term relief will report adequate relief of their symptoms for at least three of the six months between the 6- and 12-month follow-up. Otherwise, a patient will be defined as a nonresponder. Adequate relief is a validated clinically relevant endpoint and is defined at the point where the individual patient is satisfied with treatment [29].
- Quality of life will be measured with the 36-Item Short Form Health Survey (RAND-36) health survey. The RAND-36 is a valid and reliable self-reported questionnaire [30]. The questionnaire consists of eight subscales, namely physical functioning, social functioning, role-physical or emotional problems, mental health, vitality, bodily pain, and general health. A higher score on the scale of 0-100 indicates a better quality of life [30,31].
- Severity of symptoms, defined as self-perceived pain and fatigue in the past week, will be measured with an 11-point numeric scale (score 0-10) [32].
- Severity of psychosocial symptoms will be measured with the Four-Dimensional Symptom Questionnaire (4DSQ) questionnaire. This questionnaire consists of 4 subscales, namely distress, depression, anxiety, and somatization [33,34].
- Self-perceived health will be measured with the EuroQol-5D (EQ5D) questionnaire. This questionnaire will measure the perceived health on five levels (ie, mobility, self-care, usual activities, pain/discomfort, and anxiety/depression) [35].
- Physical movement behavior will be measured with the Activ8 activity monitor [36]. The Activ8 is a validated activity monitor to measure physical behavior by measuring several activities and postures (lying, sitting, standing, walking, running, and cycling). Patients will wear the Activ8 activity monitor for 1 week at varying intervals during the study. They will wear it at baseline, at 3 months follow-up, and at 12 months follow-up.
- Illness perceptions will be measured using the Brief Illness Perception Questionnaire. This questionnaire is an eight-item scale designed to assess cognitive and emotional representations of illness on an ordinal scale (0-10) [37,38].
- Self-efficacy will be measured with the Hei-Q questionnaire, which is a user friendly, valid, and reliable questionnaire specifically developed to evaluate patients’ education and self-management programs for patients with chronic complaints [39].
- Health care use and indirect costs through illness and absenteeism will be measured with Trimbos/iMTA Questionnaire for Costs associated with Psychiatric Illness (TIC-P) questionnaire to evaluate the cost-effectiveness of the program in terms of costs per Quality Adjusted Life Years (QALYs) [40]. Patients will be asked to complete the cost questionnaire every 3 months, since this questionnaire focuses on health-related costs in the past 3 months. QALYs will be measured using the EQ-5D scores [41]. In this way, we will get information of patients’ healthcare utilization and (unpaid) productivity losses.
- Besides the above parameters, the efficacy, barriers, and facilitators of the Web-based component of the blended PARASOL intervention from a patient’s perspective will be measured using the System Usability Scale (SUS). The SUS will be completed by patients of the intervention group at the end of the health care program (3-month follow-up). The questionnaire will measure the perceived usability by ten statements which can be scored on a 5-point Likert scale (‘totally agree’ to ‘totally disagree’). The SUS is a simple, valid, and reliable measurement and is often used the evaluate the usability of eHealth applications [42].

#### Other Measures

Demographic and clinical variables such as age, gender, education level, work situation, duration of complaints, and possible comorbidities will be measured at baseline. Possible comorbidities will be measured again at 3 and 12 months after baseline to determine if patients have developed comorbidities or any chronic MUPS syndromes such as fibromyalgia, chronic fatigue syndrome, or irritable bowel syndrome.

### Sample Size

The number of eligible patients was calculated according to Campbell et al for cluster randomized trials [43]. The power calculation is based on an intracluster correlation coefficient of 0.04 [44,45] and a minimum of 20 patients per health care center. Additionally, we assume a minimal clinical detectable change of >10 points in the sum score of physical functioning of the RAND-36 questionnaire, and a SD of 23.8 [10]. Based on these assumptions and a power of 80% (alpha=.05), at least ten health care centers and 206 participating patients are needed. With an expected drop-out rate of 20%, a total of 248 participating patients (124 patients per arm) are needed for the study.

**Table 1:** Summary of measures to be collected.

Outcome measures		Data collection instrument	Follow-up measurements				
			Baseline	3 months	6 months	9 months	12 months
**Primary outcome measures**							
	Impact of symptoms^a^	Adequate Relief question	✓	✓			✓
	Quality of life	36-Item Short Form Health Survey (RAND-36)	✓	✓			✓
**Secondary outcome measures**							
	Pain	Numeric Rating Scale	✓	✓			✓
	Fatigue	Numeric Rating Scale	✓	✓			✓
	Severity of psychosocial symptoms	Four-Dimensional Symptom Questionnaire	✓	✓			✓
	General health	EuroQol-5 Dimensions	✓	✓			✓
	Physical behaviour	Activ8 activity monitor	✓	✓			✓
	Illness perceptions	Brief Illness Perception Questionnaire	✓	✓			✓
	Self-efficacy	Health Education Impact Questionnaire	✓	✓			✓
	Cost-effectiveness	Trimbos and iMTA questionnaire on Costs associated with Psychiatric illness	✓	✓	✓	✓	✓
	Barriers and facilitators of the blended e-Exercise health care program	System Usability Scale		✓			
**Other measures**							
	Age	Questionnaire	✓				
	Gender	Questionnaire	✓				
	Education level	Questionnaire	✓				
	Work situation	Questionnaire	✓				
	Duration of complaints	Questionnaire	✓				
	Possible comorbidities	Questionnaire	✓	✓			✓

^a^Measured weekly between baseline and 3 months follow-up, and monthly between 6 and 12 months follow-up.

### Statistical Analysis

Statistical analysis will be performed using IBM SPSS 22. Statistical analysis will be performed according to the intention-to-treat principle. Any missing values will be imputed with the Multivariate Imputation by Chained Equations. Descriptive statistics will be used to describe the number of patients with moderate MUPS (as identified using the PRESUME screening method) which are excluded by their GPs, how many patients are recruited with the 3 different strategies, as well as how many patients do not complete the blended PARASOL intervention. Additionally, descriptive statistics (frequencies, *t*-test and chi-square test) will be used to describe the demographic characteristics of the study population and to explore baseline comparability. Differences in effectiveness of the blended PARASOL intervention will be analyzed using longitudinal mixed methods analyses. In this way, we can correct for independence of observations within patients as well as take into account possible variations between clusters and health care professionals. Analyses will be corrected for potential confounders (eg, age, gender, and psychiatric comorbidity) and potential interactions terms (eg, age in the use of the Web-based component of the PARASOL intervention) will be checked. Furthermore, the cost-effectiveness of the blended PARASOL intervention will be clarified with an incremental cost-effectiveness ratio based on the costs per QALY. All costs measured by the TIC-P (health care use and indirect costs of illness and absenteeism) are used to calculate the incremental cost-effectiveness ratio.

## Results

The components of this intervention are based on results of a literature search and focus groups with experts (general practitioners, physical therapists, mental health nurses, and psychologists) [46]**.** The content of the information, self-management, and exercise modules were specifically developed for the current study. The functionality of the online program used in this study is based on the blended exercise intervention for patients with hip or knee osteoarthritis (e-Exercise) [47].

Before the start of the intervention program, physical therapists and mental health nurses of the experimental health care centers received two days of training on the content of the blended PARASOL intervention. The training consisted of presentations on the study population, central sensitization, therapeutic neuroscience education, graded activity, and perpetuating factors for all professionals involved in the study. Furthermore, the training included discussion of the content of the online modules and instructions on their implementation. During the study, a follow-up training session for the therapists will be conducted to ensure adherence to the treatment protocol.

The recruitment of health care centers started in June 2016 and inclusion of patients began in March 2017. Follow-up assessments of patients are expected to be completed in March 2019.

## Discussion

In this randomized clinical trial, the effectiveness (including cost-effectiveness) of the PARASOL intervention, a proactive blended and integrated mental health and physical therapy intervention program, will be studied.

Although the study is well-planned and involves all relevant stakeholders, the conduction of the study will present several operational challenges. The first challenge has been identified as GPs motivation to actively participate in the recruitment of patients with moderate MUPS. Patients with MUPS are a difficult patient group for GPs and often the patient-doctor relationship is under pressure due to mismatches between the expectations of the patient and doctor [48]. To motivate GPs to recruit patients with moderate MUPS, information about the PARASOL study will be sent to them beforehand. During the study, GPs will be individually informed if one of their patients is participating in the PARASOL study. Furthermore, all participating GPs will be sent updates at 3-month intervals informing them about total patient inclusion in the study, as well as patient inclusion per GP.

A second challenge identified is the recruitment of adequate patient numbers to achieve the desired statistical power. Patients with moderate MUPS will be identified using the PRESUME screening method, following which they will be proactively approached by their GP. This proactive approach may lead to patients in a non-symptomatic phase or without a treatment demand being contacted. Consequently, these patients might be less motivated to follow the blended PARASOL intervention aiming to prevent chronicity of MUPS. To deal with this challenge, setting individual treatment goals has been identified as an important part of the intake session. It should be noted that the face-to-face sessions are not performed on a weekly basis to not only reduce the burden for patients, but more importantly to encourage self-management.

A third challenge is the potential drop-out rate in the control group since these patients will not be receiving the blended PARASOL intervention and therefore may be less motivated to participate in the study. To deal with this challenge, patients in the control group will be offered to follow the blended PARASOL intervention after the study ends.

A final identified challenge is the non-usage of the Web-based component of the blended PARASOL intervention. Previous studies have shown that patients in online interventions are less motivated and feel less pressure to continue with the intervention compared to face-to-face interventions [49]. To combat this, patients will receive email reminders for the eHealth modules weekly. Furthermore, the PARASOL intervention has been designed as a blended care program, and this is therefore expected to maximize adherence compared to self-guided internet interventions [50].

Besides these challenges, there are several strengths and limitations in the design of the study that should be noted. The first strength of this study is that physical therapists and mental health nurses will participate in two days of intensive training about the content of the blended PARASOL intervention. This will minimize the differences in the care offered by professionals at different health care centers during the health care program [51]. In addition, a meeting with the participating physical therapists and mental health nurses will be organized after 6 months to discuss the content of the blended PARASOL intervention as well as any possible difficulties faced. The 12-month follow-up measurement is another strength of this study as it will result in data being obtained about long-term effectiveness (and cost-effectiveness) of the program. The PARASOL intervention stimulates self-management by focusing on achieving a healthier lifestyle as well as the adoption and maintenance of exercise behavior. Since the process of adopting a change to maintaining a change takes at least six months, a long-term follow-up is of particular interest [52]. A third strength of this study is performing cluster randomization at the level of the health care centers as this ensures that a contamination-effect will be avoided [22]. Finally, this is the first study, to the best of our knowledge, that investigates the effectiveness of an intervention program for patients with moderate MUPS to prevent chronicity.

The first identified limitation of this study, is that it is unblinded. Patients, health care professionals, and the researchers are aware all of the group allocated to the blended PARASOL intervention. This may lead to bias mechanisms such as response bias or observer bias being present in the data [53]. One of the aims of the training provided to the healthcare professionals involved in the study is to avoid response bias from the health care professionals. Observer bias will be avoided by using a measurement protocol, well trained observers, and standardized outcome measures. A second limitation is that overtreatment may occur since not all patients with moderate MUPS will be prevented from developing chronic MUPS after completing the PARASOL intervention. This could lead to higher health care costs if patients are still consulting health care professionals after completing the PARASOL intervention. However, an early intervention for patients with moderate MUPS may lead to a decrease of direct and indirect costs on long term if chronic MUPS is prevented. Therefore, one of the secondary objectives is to determine the cost-effectiveness of the PARASOL intervention. A third limitation is complexity of the design of the study due to the use of cluster randomization. Cluster randomized trials are more complex, require more patients to obtain equivalent statistical power, and require more complex analysis [43]. However, in the sample size calculation and statistical analysis, this possible design effect has been taken into account.

This study is the first trial that investigates the effectiveness (including cost-effectiveness) of a blended care program in patients with moderate MUPS. Therefore, this study will provide relevant results regarding short- and long-term effectiveness of a multidisciplinary, blended care program to prevent chronic MUPS.

## Appendix

Multimedia Appendix 1 Schematic view of the blended health care program.

## Abbreviations

4DSQ: Four-Dimensional Symptom Questionnaire
EQ5D: EuroQol-5D
GP: general practitioner
ICPC: International Classification of Primary Care
MUPS: medically unexplained physical symptoms
PRESUME: preventive screening of medically unexplained physical symptoms
RAND-36: 36-Item Short Form Health Survey
TIC-P: Trimbos/iMTA Questionnaire for Costs associated with Psychiatric Illness
QALY: Quality Adjusted Life Years
