# Supplementary material for: Effectiveness of a Blended Multidisciplinary Intervention for Patients with Moderate Medically Unexplained Physical Symptoms (PARASOL): Protocol for a Cluster Randomized Clinical Trial
Source: JMIR Res Protoc. 2018 May 8;7(5):e120. doi: 10.2196/resprot.9404 (PMC5964304; doi:10.2196/resprot.9404)
Supplement: Multimedia Appendix 1 [file resprot_v7i5e120_app1.pdf]

Schematic view of the blended health care program

Intake	Physical therapist	Anamnesis and physical examination<br>Providing information about the web-based part of the health care program
	Mental health nurse	Anamnesis according to the SCEGS
Week 1	Physical therapist	Education about therapeutic neuroscience education<br>Providing information about the 3-day baseline self-test
	Mental health nurse	Education about perpetuating factors
	Web-based component	Module 1: Central sensitization & perpetuating factors<br>Performance of a 3-day baseline test
Week 2	Web-based component	Module 2: Graded activity & Behavioral change
Week 3	Physical therapist	Evaluation of education week 1<br>Education about graded activity<br>Evaluation results from the 3-day self-test<br>Determining short term goal<br>Discussing the gradual increase of the selected activity
	Mental health nurse	Patient specific inventory on perpetuating factors<br>Education about coping strategies
	Web-based component	Starting gradually increase selected activity
Week 4	Web-based component	Module 4: Stress
Week 5	Web-based component	Module 5: Coping with physical complaints
Week 6	Physical therapist	Evaluation of graded activity<br>Evaluation of online modules<br>Coaching on lifestyle
	Mental health nurse	Evaluation of perpetuating factors<br>Evaluation of the coping strategies
Week 7	Web-based component	Module 7: Relaxation
Week 8	Web-based component	Module 8: Lifestyle
Week 9	Web-based component	Module 9: Creating an exercise plan
Week 10	Web-based component	Module 10: Performing the exercise plan
Week 11	Web-based component	Module 11: Maintaining an active lifestyle and avoiding a relapse
Week 12	Physical therapist	Discussing long-term goals<br>Support to maintain a physically active lifestyle
